# *Haematospirillum jordaniae* Infections after Recreational Exposure to River Water, Pennsylvania, USA, 2020

**DOI:** 10.3201/eid3111.241586

**Published:** 2025-11

**Authors:** Melissa Dulcey, Katherine M. DeBord, Melissa E. Bell, Meghan T. Murray, Adam M. Szewc, Kate Livingston, Brendan Headd, John R. McQuiston, Eva Gordian-Rivera, Ben W. Humrighouse, Allison Longenberger, William A. Bower

**Affiliations:** Centers for Disease Control and Prevention, Atlanta, Georgia, USA (M. Dulcey, K.M. DeBord, M.E. Bell, M.T. Murray, A.M. Szewc, K. Livingston, B. Headd, J.R. McQuiston, B.W. Humrighouse, W.A. Bower); Pennsylvania Department of Health, Harrisburg, Pennsylvania, USA (M. Dulcey, M.T. Murray, E. Gordian-Rivera, A. Longenberger)

**Keywords:** *Haematospirillum jordaniae*, communicable diseases, emerging infections, bacterial infection, bacteria, fresh water, Pennsylvania, United States

## Abstract

*Haematospirillum jordaniae* was first identified as a human pathogen in 2016. In this article, we describe 4 patients who had *H. jordaniae* infections identified in 2020 and who had temporally and spatially linked environmental exposures. Three of the 4 patients reported leg injuries while participating in recreational river water activities in south-central Pennsylvania, USA. In 2024, we detected *H. jordaniae* in river samples collected at locations identified during patient interviews. All patients sought emergency department services for clinical assessment; however, the causative bacterial isolate was not initially identified. *H. jordaniae* was identified as the bacterial cause months after patient treatment and discharge. Although *H. jordaniae* infections are considered rare, the true occurrence is unknown. Additional information about the organism’s ecology and environmental seasonality could guide public health messaging and increase awareness among healthcare providers.

*Haematospirillum jordaniae* is a slow-growing, gram-negative, rod-shaped bacterium that belongs to the alphaproteobacteria family Rhodospirillaceae*. H. jordaniae* was first identified as a human pathogen in 2016 by the Special Bacteriology Reference Laboratory (SBRL) at the Centers for Disease Control and Prevention (CDC) (*1*). SBRL identified 25 total *H. jordaniae* isolates that were sent from state health departments across the United States during 2000–2019. All 25 historical isolates were obtained from blood samples collected from adult men averaging 58 (range 39–78) years of age. Most of the clinical cases were identified during summer and fall months, suggesting a seasonal trend associated with *H. jordaniae* infection. In this article, we describe 4 patients who had *H. jordaniae* infections identified in 2020 and who had environmental exposures in a defined region of Pennsylvania, USA. We also describe the results of environmental testing for *H. jordaniae* conducted in the same area in 2024.

## Methods

### Identifying the Bacteria in Clinical Samples

Patient blood samples were sent to the Pennsylvania Department of Health (PADOH)’s Bureau of Laboratories for bacterial identification and to rule out *Brucella* species, according to standard Laboratory Response Network protocols. After negative *Brucella* results and the inability to identify the isolates, bacterial isolates were then sent to SBRL at CDC for bacterial identification. We identified the bacterial isolates recovered from each patient using previously described methods for16S rRNA gene sequence analysis and Clinical Laboratory and Standards Institute guidelines (*1*–*3*). We also used matrix-assisted laser desorption/ionization time-of-flight (MALDI-TOF) analysis as a secondary identification method. We performed MALDI-TOF analysis on a Biotyper SMART (Bruker) by using an in-house library containing reference spectra from 6 strains of *H. jordaniae*, which is publicly searchable through MicrobeNet (https://microbenet.cdc.gov). All isolates yielded a MALDI-TOF score of >2.0 for a reliable species-level identification as *H. jordaniae*.

### Medical Information

We conducted emergency department (ED) medical chart reviews for all 4 patients. Public health officials conducted interviews with patients 1, 2, and 3. Patient 4 could not be reached for interview.

### Environmental Sample Collection

We identified 3 water locations in Pennsylvania for environmental sampling on the basis of patient interviews. Patient 2 identified locations A and B as their entry and exit points in the creek on the day of injury, near where patient 1 also reported injury. Patient 3 identified location C as the location where the patient entered the river on the day of injury, ≈50 miles from locations A and B.

We collected four 1-L water samples from locations A and B once a month throughout 2024 and four 1-L water samples from location C for 7 months in 2024. We collected water samples from the river’s surface, enabling the current to flow into the container without disturbing the river bottom sediment. At each sample collection site, we collected global positioning satellite coordinates, weather conditions, and water physiochemical measurements (pH, temperature, dissolved oxygen, conductivity, and specific conductance) by using a YSI Pro 2030 (YSI Incorporated).

### Environmental Sample Processing and Testing

We shipped water samples to SBRL on cold packs on the day of sample collection. Because of resource constraints, a subset of the total number of collected samples were processed for DNA extraction. We filtered 1-L samples through MicroFunnel filter funnels containing a 0.22 μm filter (Cytiva), and we extracted genomic DNA from the filters by using the DNeasy PowerWater Kit (QIAGEN) according to manufacturer instructions. We analyzed extracted filter genomic DNA by using previously published methods for a real-time quantitative PCR (qPCR) specific to *H. jordaniae* (*4*). Modifications of methods for the screening of the submitted water samples included using the previously published qPCR with environmental samples and testing environmental genomic DNA extracted from water filters. We used 2 layers of controls, including positive (PCR amplification) and negative (nontemplate) controls for qPCR, as well as positive and negative filter extraction controls, which aided in confirming that targeted DNA could be collected through experimental filter extraction.

### Ethics

This study did not meet the definition of human subjects research as defined in the US Code of Federal Regulations, Title 45 Part 46. Therefore, this study was not subject to review by an institutional review board.

### Case Series

#### Patient 1

**Figure 1:**
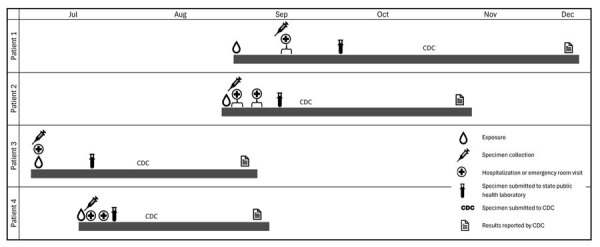
Clinical timeline of 4 patients with *Haematospirillum jordaniae* infections, Pennsylvania, USA, 2020. Three of the 4 cases were later determined to be associated with recreational freshwater exposure.

Patient 1 was a 28-year-old man who reported a scratch to his right distal leg in September 2020 (Figure 1). The injury occurred while participating in a recreational water activity in a south-central Pennsylvania creek. Within 24 hours of the injury, redness developed on the medial aspect of the patient’s right lower extremity. Two days later, swelling and pain developed at the location of the redness. The patient was initially treated by his primary care physician by telehealth appointment and prescribed cephalexin. After treatment was initiated, the redness started to improve but then became worse and spread down the patient’s leg, resulting in swelling of his ankle. The patient denied any fever or chills and was taking oral cephalexin (500 mg every 6 h for 10 days) as prescribed.

Ten days after the initial injury, the patient sought care at a local ED with a primary complaint of severe pain associated with the right lower extremity wound infection. Temperature was 37.1°C, heart rate 115 beats/min, respiratory rate 18 breaths/min, blood pressure 148/102 mm Hg, and oxygen saturation 99% on room air. Physical examination revealed swelling, tenderness, and erythema on the medial aspect of the right lower extremity from the ankle to halfway up the tibia. The patient’s remaining physical examination findings were unremarkable. Laboratory results at admission revealed an elevated leukocyte count of 21,800 cells/μL (reference range 3,900–9,500 cells/μL) (Table).

**Table:** Laboratory results during initial hospital admission of 4 patients with *Haematospirillum jordaniae* infections with recreational water exposure in 3 of 4 cases, Pennsylvania, USA, 2020*

Laboratory values	Patient 1	Patient 2	Patient 3	Patient 4
Leukocyte count, cells/μL	21,800 (3,900–9,500)	24,200 (3,900–9,500)	22,430 (4,000–10,800)	21,000 (4,000–11,000)
Neutrophil count, cells/μL	18,300 (1,800–7,400)	20,570 (1,800–7,400)	17,620 (1,800–7,700)	18,000 (1,700–7,800)
Platelets/μL	394,000 (140,000–366,000)	413,000 (140,000–366,000)	247,000 (140,000–400,000)	265,000 (140,000–400,000)
Creatinine, mg/dL	0.87 (0.7–1.3)	0.75 (0.6–1.2)	0.8 (0.6–1.2)	0.91 (0.7–1.3)
Hemoglobin, g/dL	15 (12.8–16.6)	13 (11.7–15.1)	15.3 (14.0–16.8)	14.3 (13–17.3)
Calculated eGFR non-African American, mL/minute/1.73 m^2^	117 (>90)	104 (>89)	>60 (>60)	>90.0 (>90)

*Values in parentheses are hospital laboratory reference values. eGFR, estimated glomerular filtration rate.

The patient was given 1 dose of vancomycin (1.25 g) in the emergency room before being hospitalized for sepsis and cellulitis and started on intravenous (IV) vancomycin (1.5 g every 8 h) and IV clindamycin (600 mg every 8 h) antimicrobial therapy. Radiographs and ultrasound of the right ankle showed a moderate amount of soft tissue swelling surrounding the ankle that was consistent with cellulitis. No fluid collection was observed in the ankle to indicate a septic joint, and no radiopaque foreign body or acute osseous abnormalities were observed. The patient clinically improved, was discharged after 3 days of in-hospital treatment, and was prescribed a 7-day course of oral clindamycin (300 mg every 6 h). A blood sample obtained at admission was cultured, and a bacterial isolate was recovered; 16S rRNA gene sequence analysis showed the isolate to be most closely related to *H. jordaniae* (reference strain no. DSM 28903^T^). A nearly full-length (1,406-bp) 16S rRNA gene sequence from the isolate shared 99.9% sequence similarity with DSM 28903^T^. On the basis of Clinical and Laboratory Standards Institute guidelines (*3*), we identified the isolate (GenBank accession no. PX060169) as *H. jordaniae* 3 months after the patient’s admission.

#### Patient 2

Patient 2 was a 34-year-old woman with a history of rheumatoid arthritis, hypothyroidism, idiopathic thrombocytopenic purpura, hypertension, and migraines. She reported hitting her left shin on a rock in August 2020 while participating in a recreational water activity in a south-central Pennsylvania creek (Figure 1). The patient reported redness and discomfort developing in the area the day after injury and severe pain of the lower leg around superficial abrasions on the left shin that developed 3 days later. The patient subjectively reported low-grade fever, without objective temperatures >100°F (37.8°C). Four days after the initial exposure, the patient was evaluated by her primary care physician, who was concerned that compartment syndrome might have developed and advised her to go to the ED for further evaluation.

During ED examination, the patient reported severe left lower leg pain. Temperature was 37.7°C, heart rate 121 beats/min, respiratory rate 18 breaths/min, blood pressure 150/108 mm Hg, and oxygen saturation 100% on room air. Physical examination revealed erythema and tenderness of the left lower extremity, and a 1 cm abrasion was located on the anterior aspect of the leg distal to the left knee. Edema of the left lower leg was also noted. The rest of the physical examination was unremarkable. Laboratory results at admission revealed an elevated leukocyte count of 24,200 cells/μL (reference range 3,900–9,500 cells/μL) (Table).

The patient was hospitalized for sepsis attributable to cellulitis and administered a single dose of IV ceftriaxone (1 g) in the ED and started on IV cefazolin antimicrobial therapy (2g every 8 h) after admission as treatment for potential group B *Streptococcu*s infection. Radiographs of the patient’s left lower fibula and tibia showed subcutaneous phleboliths (calcification of blood clots) likely unrelated to the injury and no radiopaque foreign body or acute osseous abnormalities. The patient clinically improved during hospitalization with decreased left leg pain, improved leukocyte count, and improved physical therapy evaluations. She was discharged after 2 days and prescribed an 8-day course of oral cephalexin (500 mg 4×/d).

Within 2 days of discharge, the blood cultures performed at admission were positive for unspecified gram-negative rod-shaped bacteria, and the patient was advised to return to the ED for evaluation. At admission, the patient reported improved symptoms with decreased redness and swelling at the site and an increased ability to ambulate on her left leg. She denied any fevers, chills, nausea, or vomiting. On physical examination, a slight warmth around the healing abrasions was noted, the erythema on their left leg had improved, and no edema was apparent. Repeat blood culture, lactic acid, basal metabolic panel, and complete blood count were performed. Other than a slightly elevated leukocyte count at 9,600 cells/μL (reference range 3,900–9,500 cells/μL), other values were unremarkable. The patient was hospitalized and treated with IV cefepime (2 g every 8 h); while hospitalized, the patient continued to clinically improve. She was discharged on day 3 with a 10-day course of oral levofloxacin (750 mg/d). The blood cultures from blood obtained during the second hospitalization were negative. Two months later, we identified the isolate from a blood sample obtained during the patient’s initial hospitalization as *H. jordaniae* (GenBank accession no. PX060171). A nearly full-length (1,408-bp) 16S rRNA gene sequence from the isolate shared 99.9% sequence similarity with DSM 28903^T^.

#### Patient 3

Patient 3 was a 36-year-old man with history of diabetes mellitus, obesity, and boils. He reported hitting his leg in July 2020 while participating in a recreational water activity in a south-central Pennsylvania river (Figure 1). The patient sought care at the ED the day after the injury with pain, swelling, and erythema of the anterior aspect of the right leg.

At initial examination, the patient reported severe right lower leg pain. Temperature was 36.4°C, heart rate 106 beats/min, respiratory rate 16 breaths/min, blood pressure 164/76 mm Hg, and oxygen saturation 97% on room air. Physical examination showed a 10.5 cm area of poorly defined erythema, warmth, and tenderness of the anterior aspect of the right leg. No drainage, breakage of the skin, or crepitus of the limb’s joints was noted. Laboratory results at admission demonstrated an elevated leukocyte count of 22,430 cells/μL (reference range 4,000–10,800 cells/μL) (Table).

The patient was administered a single dose of IV clindamycin (800 mg) for presumed bacterial infection. Radiographs of the right tibia and fibula showed chronic calcification of the diaphysis of the tibia, unchanged from a prior radiograph, and no signs of acute osseous abnormalities, radiopaque foreign body, or soft tissue gas. On the basis of the patient’s age, comorbidities, laboratory results, imaging, and examination findings, the decision to treat as an outpatient with a primary care physician follow-up was made. The patient was discharged with a 10-day course of oral clindamycin (300 mg every 8 h).

Within 4 days of discharge, the blood culture performed at admission was positive for gram-negative rod-shaped bacteria. The patient was advised to return to the ED for evaluation and additional blood cultures. At admission, the patient reported feeling better with right leg improvement. He had slight tenderness and itchiness of the right leg, with pain only when walking and during palpation. Physical examination showed an increased warmth of the right anterior lower leg compared with the left leg and a central scabbed-over pinpoint wound on the right leg with mild erythema. Bloodwork revealed resolved leukocytosis; other blood values, radiographs, and urinalysis results were unremarkable. The patient was hospitalized and received IV cefepime (dose unknown) for bacteremia and cellulitis of the right lower extremity. While hospitalized, the patient clinically improved, and the redness of the right leg completely resolved. Three days after collection, preliminary results for the repeat blood cultures were negative. After consultation with an infectious disease doctor, the patient was discharged on a course of oral ciprofloxacin (500 mg every 12 h) and oral amoxicillin/clavulanate (500 mg/125 mg every 8 h) (length of course unknown). The blood cultures obtained during hospitalization were negative after 5 days of incubation. Gram-negative rod-shaped bacteria were recovered by the hospital laboratory from blood cultures collected at the patient’s initial ED visit. We identified the isolate as *H. jordaniae* 1.5 months after the patient’s discharge (GenBank accession no. PX060170). A nearly full-length (1,408-bp) 16S rRNA gene sequence from the isolate shared 99.9% sequence similarity with DSM 28903^T^.

#### Patient 4

Patient 4 was a 48-year-old man with history of fatty liver who sought care at the ED in July 2020 with a history of a fever up to 101°F (38.3°C), nausea, and left lower leg erythema with swelling of 1-day duration (Figure 1). The patient reported being at a cabin the prior 2 days and noticed bug bites and scratches develop on his legs while walking in shorts. Additional information about any water-related exposures for this patient are unknown as the patient was unable to be reached for an interview.

At initial examination, the patient had a fever of 38.6°C; heart rate was 111 beats/min, blood pressure 107/67 mm Hg, and an oxygen saturation of 97% on room air. Physical examination showed bug bites and erythema on the anterior distal shin of the left leg that did not extend to the knee or thigh. Mild edema was also noted in areas of erythema. Tachycardia was the only other major physical examination finding. Laboratory results at admission revealed an elevated leukocyte count of 21,000 cells/μL (reference range 4,000–11,000 cells/μL) (Table).

The patient was observed in hospital for cellulitis and administered a single dose of IV cefazolin (2g). Ultrasound of the left lower extremity revealed no signs of deep vein thrombosis. The patient’s fever and tachycardia improved with IV fluids, ketorolac tromethamine, and acetaminophen. The patient was discharged ≈4 hours after admission with a 7-day course of oral sulfamethoxazole/trimethoprim (800 mg/160 mg every 12 h). Two months later, we identified an isolate from a blood sample obtained during the patient’s ED treatment as *H. jordaniae* (GenBank accession no. PX060172). A nearly full-length (1,408-bp) 16S rRNA gene sequence from the isolate shared 99.9% sequence similarity with DSM 28903^T^.

### Environmental Testing

**Figure 2:**
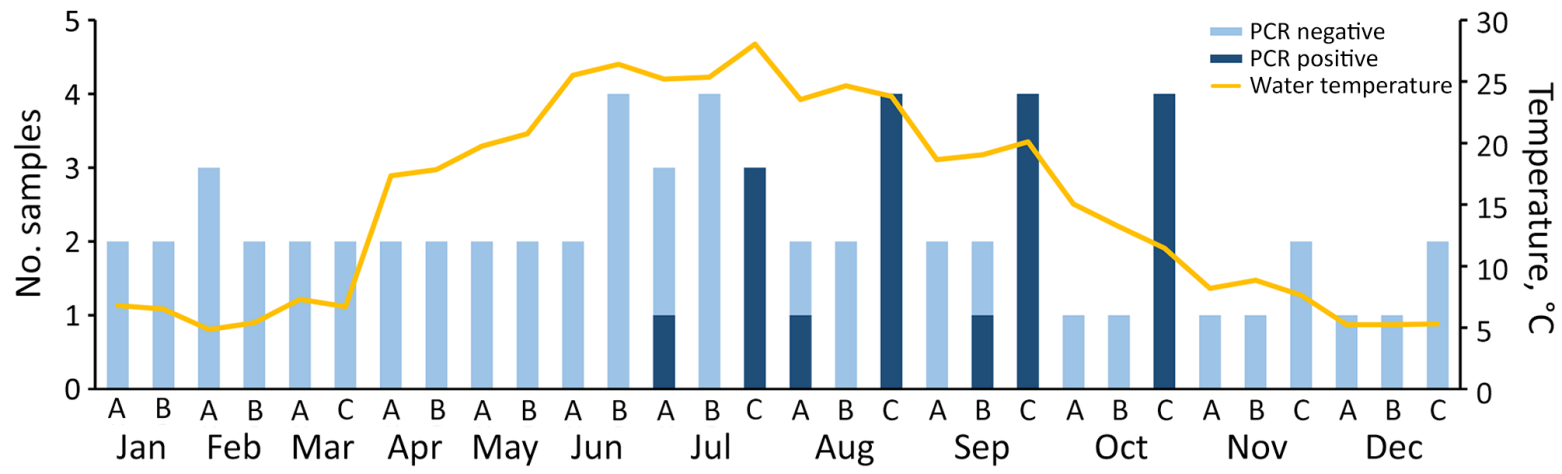
Environmental testing results for *Haematospirillum jordaniae* and water temperature measurements at 3 locations (A–C) in Pennsylvania, USA2024, in study of 4 patients with *H. jordaniae* infections from 2020 after recreational water exposure.

We collected 121 total water samples. We processed and extracted DNA from 67 (55%) samples: 23 from location A, 23 from location B, and 21 from location C. Of the 67 processed samples, 18 (27%) tested positive for *H. jordaniae* by qPCR. All positive samples were collected during July–October; 83% (n = 15) of positive samples were from location C, but all 3 sampling sites had >1 positive sample (Figure 2). During July–September, location C demonstrated an average cycle threshold value difference of 5.88 compared with locations A and B, representing almost a 2-log increase in bacterial load at location C compared with the other sampling locations. Water temperature ranged from 4.9° to 28.1°C throughout the year, with peak temperatures recorded in July and August.

## Discussion

We identified 4 patients with *H. jordaniae* infections that were spatially and temporally linked in Pennsylvania in 2020. Through systematic environmental sampling over a 1-year period in 2024, we identified *H. jordaniae* in the implicated water sources for 3 of the 4 patients at the same time of year when the patients’ exposures occurred.

Three of 4 patients reported leg injuries and freshwater exposure. Exposures occurred independently of one another while participating in recreational water activities in waterways that are tributaries of the Susquehanna River in south-central Pennsylvania. The fourth patient’s exposure to freshwater is unknown; however, he did spend time outdoors during the same period. A handful of sporadic cases of *H. jordaniae* have been published previously (*5*–*7*), including 2 persons with documented freshwater exposure accompanied by an injury to the skin or soft tissue, similar to 3 patients reported in this article (*5*). Although we have limited epidemiologic details on *H. jordaniae* isolates submitted to CDC, most historical specimens were collected during July and August, which coincides with the timing of infections for the 4 case-patients presented in this article. 

Environmental testing over a 1-year period at 3 locations where the patients reported entering or exiting the rivers suggest seasonality in the detection of *H. jordaniae*. All positive environmental samples were collected during July–October, coinciding with the timing of the 4 patients’ illnesses, as well as historical *H. jordaniae* infections. Despite those findings, information on the presence and persistence of *H. jordaniae* in the environment remains limited. Although this study is a systematic sampling and testing for *H. jordaniae* in the environment in Pennsylvania, the study was limited in scope. Because of limited resources, we were not able to process all samples collected or visit location C for all months of the year. The full geographic distribution of *H. jordaniae* in this area and its persistence in water remains unknown. Additional information about the organism’s ecology and seasonality in the environment can guide future messaging efforts focused on disease prevention and increase awareness of the pathogen’s transmission among healthcare providers and the public.

Although *H. jordaniae* infections are considered rare, the true occurrence is unknown. For the 4 patients in this cluster, the final identification of *H. jordaniae* as the bacterial cause of their infections did not occur until months after they were treated and discharged. Diagnosing an *H. jordaniae* infection can be challenging because of the difficulty in recovering the bacteria from primary specimens because of the slow growth and fastidious nature of the organism. Bacterial identification was attempted at the hospital or the PADOH laboratories before isolates were submitted to CDC, leading to additional logistical delays in final identification. Recently, laboratory methods for a sensitive, specific, and efficient real-time qPCR assay were published (*4*). Such methods could be used by state and local health departments to decrease time to diagnosis and to identify cases of *H. jordaniae* that would otherwise go undetected if specimens are not submitted to CDC. Taxonomic updates to bacterial databases for commercial platforms might also improve identification in hospital and state laboratories, decreasing the time for identification.

Information regarding *H. jordaniae* and its effects on human health remains limited. Although *H. jordaniae* infections are rare, this case series highlights freshwater exposure as a potential route of transmission. Clinicians should be vigilant in identifying bacterial infections and the potential for sepsis during midsummer and early fall when treating skin wounds that might be associated with freshwater exposure. In addition, *H. jordaniae* should be considered in the differential diagnosis for those patients when requesting bacterial identification from local public health laboratories. This request could expedite bacterial identification and provide a better estimate of the total number of *H. jordaniae* infections across the United States.
